# Rapid corn and soybean mapping in US Corn Belt and neighboring areas

**DOI:** 10.1038/srep36240

**Published:** 2016-11-04

**Authors:** Liheng Zhong, Le Yu, Xuecao Li, Lina Hu, Peng Gong

**Affiliations:** 1 Department of Environmental Science, Policy and Management, University of California, Berkeley, California, USA; 2 Ministry of Education Key Laboratory for Earth System Modeling, Center for Earth System Science, Tsinghua University, Beijing, 100084, China; 3 Joint Center for Global Change Studies, Beijing 100875, China; 4 Department of Geological & Atmospheric Sciences, Iowa State University, Ames, Iowa, 50011, USA; 5 Department of Sociology, Tsinghua University, Beijing 100084, China

## Abstract

The goal of this study was to promptly map the extent of corn and soybeans early in the growing season. A classification experiment was conducted for the US Corn Belt and neighboring states, which is the most important production area of corn and soybeans in the world. To improve the timeliness of the classification algorithm, training was completely based on reference data and images from other years, circumventing the need to finish reference data collection in the current season. To account for interannual variability in crop development in the cross-year classification scenario, several innovative strategies were used. A random forest classifier was used in all tests, and MODIS surface reflectance products from the years 2008–2014 were used for training and cross-year validation. It is concluded that the fuzzy classification approach is necessary to achieve satisfactory results with R-squared ~0.9 (compared with the USDA Cropland Data Layer). The year of training data is an important factor, and it is recommended to select a year with similar crop phenology as the mapping year. With this phenology-based and cross-year-training method, in 2015 we mapped the cropping proportion of corn and soybeans around mid-August, when the two crops just reached peak growth.

Corn and soybeans are two of the most important agricultural commodities in the world^1^,^2^. Global crop trading activities and market prices are very sensitive to information on production before or during the growing season. As the largest cultivation area of corn and soybean, the US Corn Belt and neighboring states (called the Extended Corn Belt or ECB) account for about one third of global production, according to statistical data from the Food and Agriculture Organization. (http://faostat3.fao.org/home/E). For this area, numerous studies and intensive networks have been established to make production forecasts based on surveys, census, fieldwork and statistical analysis by governmental and private sectors. Remote sensing provides objective means to efficiently estimate cropland extent^3^,^4^ and crop production^5^, and remote sensing-based agricultural assessment has become a regular effort for the ECB^6^,^7^,^8^,^9^,^10^,^11^. However, compared with existing fast-response, survey-based approaches, it is challenging for remote sensing studies to meet practical requirements on a timely basis, owing to limitations of image and reference data availability, processing time, and other factors. The goal of obtaining early information has become one of the foci of remote sensing-based crop monitoring^12^,^13^.

In general, corn or soybean production is calculated as yield multiplied by harvested area. Remote sensing-based yield models have been built based on the statistical relationship between multispectral observations and yield. For estimates of harvested area, image classification is important to mapping the extent of corn and soybean fields. Successful and accurate mapping of crop extent is also critical to yield estimation, because including pixels of the wrong type can bias the performance of statistical models^5^,^13^. Therefore, early mapping of crop extent with high accuracy is essential for reliable production forecasts within the growing season.

To ensure timeliness of the crop map, the rapid mapping approach needs to meet the following practical requirements.

- The approach should be able to use reference data of previous years for training. For a large area like the ECB, field data collection is extremely time-consuming, expensive, and labor-intensive. At present, existing reference data and other datasets of comparable quality are usually unavailable to the general public within the growing season. Actual crop-type data reported to the USDA Farm Service Agency are confidential and for only internal use of the USDA. The USDA National Agricultural Statistics Service (NASS) Cropland Data Layer (CDL), a georeferenced raster map of specific crop types, is not released until the beginning of the subsequent calendar year for market sensitivity reasons. All these facts suggest that the only way to quickly collect training data for the entire ECB is to use reference data from previous years. Because the training year and mapping/validation year are different, this type of classification is called cross-year mapping or cross-year validation.
- The algorithm needs to be capable of handling interannual variability in crop progress, accounting for varying phenological development. Because the classifier is trained by reference data and remotely sensed time series from a different year, the classification result may not be satisfactory, especially when there is a tremendous difference in crop progress between the training and mapping years. As a solution, the cross-year mapping approach must be able to make use of crop growth information as ancillary datasets in the classification, in order to quantify interannual variability of crop progress.

Most studies on corn and soybean classification have either focused on single-year mapping or ignored the difference in crop progress between the training and mapping years. Traditionally, time series of multi-spectral observations or vegetation indices are the main input variables to the classification algorithm^7^,^8^,^14^. However, for cross-year mapping, the imaging date that yields the best separation in one year may be useless in another year. Differences in image quality, cloud cover and other factors across years may also increase the difficulty of cross-year mapping. Phenology-based approaches have used metrics related to crop phenology, such as emergence and maturity dates, to improve the robustness of multi-temporal classification^9^,^11^,^13^,^15^,^16^,^17^,^18^. Compared with original image bands and vegetation indices on certain dates, phenological metrics directly represent crop progress and growing conditions that are comparable to physical properties and field experience^19^,^20^. Although timeliness is not the focus of most existing phenology-based classification efforts, phenological metrics provide the ability to facilitate cross-year mapping by quantitatively measuring interannual variability of crop phenology.

The USDA provides a variety of datasets regarding cropping extent and progress in the ECB. The USDA CDL is a georeferenced raster map of specific crop types, produced using satellite imagery from multiple sensors^8^. For corn and soybean extent in the ECB, the CDL generally has very high accuracies (greater than or around 95% for major production areas according to metadata; available at https://www.nass.usda.gov/Research_and_Science/Cropland/metadata/meta.php) that are almost equivalent to reference data for training^9^,^13^. USDA crop production statistics including corn and soybean acreage are produced from a variety of surveys and the Census of Agriculture facilitated by the NASS. As a traditional response-based product, statistics of cropped acreage offer an independent dataset to validate classification results. The USDA Crop Progress and Condition Report (CPCR) provides crop development stage percentages on a weekly basis, which are a valuable source of phenological measurements that may be comparable to remotely detected crop phenology. These three USDA products offer crop production data of different aspects and show great potential to support crop classification, such as improving the accuracy and timeliness of crop maps. However, few studies have fully used these products in ECB corn and soybean mapping.

The goal of this study is to accurately and quickly map the extent of corn and soybeans in the ECB. To meet the time requirement of crop mapping in practice, we applied a rapid mapping approach to finish mapping by two dates, one after the full growing season and the other at peak growth before the harvest. We also obtained reliable county-level estimates of cropped area by both dates.

## Materials and Method

### Study area

**Figure 1:**
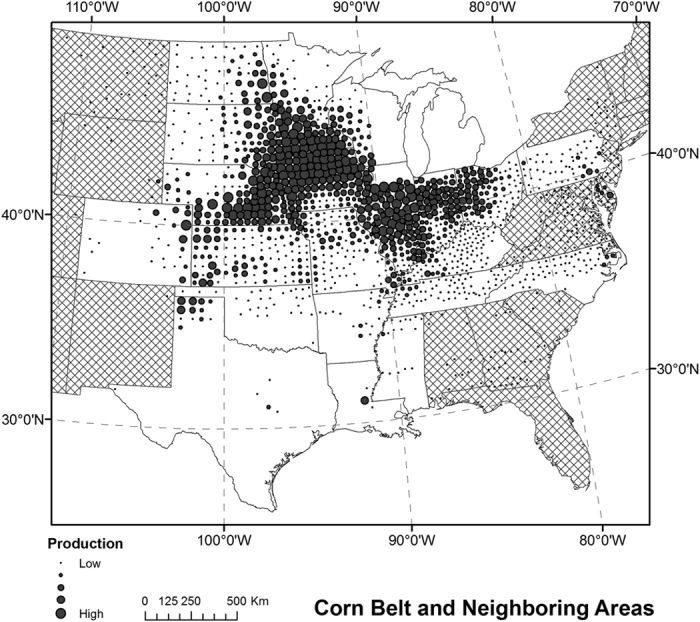
The study area, including states within or around the US Corn Belt with corn and/or soybean production (unshaded states). County-level corn production is represented by circles of various sizes to delineate the general distribution of cropland. The map was generated by ArcGIS 10.3 software (http://www.esri.com/software/arcgis/arcgis-for-desktop).

The study was conducted in 22 states within or around the US Corn Belt (Fig. 1), which is collectively called the ECB. All these states are characterized by a certain level of agricultural development with corn and/or soybean production, which is treated in USDA weekly reports of crop progress. This is a vast area with great diversity of climate, topography, and other conditions. The climate is humid continental in the northern part, to humid subtropical in the south, to cold and semiarid in the west. Annual precipitation ranges from ~1,700 mm in the gulf area of Louisiana and Mississippi, to less than 250 mm in mountainous areas of the western states. The flat area in the central ECB is very suitable for mechanized agricultural production, whereas boundary regions like the Rocky Mountains and Appalachian Mountains restrict cropland expansion.

There are a variety of crop type map products available for the ECB like the USDA CDL^8^, global land cover maps with specific crop types^2^,^4^, and regional cropland maps^6^,^9^,^13^. Because of abundant land cover data, the ECB is usually not considered a top-priority area for crop mapping^21^, but is an ideal study area for multiyear experiments. During the development of our approach, an area with substantial data availability like the ECB was preferred for cross-year mapping and validation.

### Data

#### MODIS

The primary input data were from the MODerate-resolution Imaging Spectroradiometer (MODIS) product MCD43A4, nadir and bidirectional reflectance distribution function adjusted spectral reflectance bands^22^. MCD43A4 reflectance is computed using daily Terra and Aqua satellite overpasses within each 16-day window, using an algorithm based on the inversion of radiative transfer models. The algorithm adjusts reflectance at local solar noon, reducing the effects of view angle and anisotropic scattering. The MCD43A4 product includes MODIS spectral bands 1–7 at 500-m resolution, every 8 days. A detailed set of quality information product MCD43A2 is associated, which was used to exclude low-quality observations. In general, MCD43A4 provides robust and reliable reflectance time series with acceptable resolution and frequency, showing great potential for large-scale mapping.

#### USDA CDL

The CDL is a raster, georeferenced, crop-specific land-cover map created for the continental US, using satellite imagery and extensive agricultural ground truth. Since 2008, the CDL program has provided cropland area estimates and digital products of spatial distribution for all states in the ECB, on an annual basis. All historical CDL products are available for use and are free for download through the USDA web portal. The CDL release date is usually at the beginning of the subsequent calendar year, for example, the 2014 CDL was released on 2 February 2015, timed with the release of official county estimates.

In the ECB, the CDL corn and soybean extent has a very high quality, with self-reported producer’s and user’s accuracies mostly greater than or around 95%. Because no geo-referenced data from census or survey across the entire ECB have been released to the public, annual crop-specific maps by the CDL program were used as imperfect but very reliable reference data. As the focus of the present study, corn and soybean pixels were extracted from the CDL, and all other land-cover types were merged into a class called “other”.

#### USDA crop progress

Every week in the growing season, the USDA NASS releases the CPCR, listing progress of cropping stages and overall condition of selected crops in major producing states. In the CPCR, corn progress is estimated for the “planted”, “emerged,” “silking,” “dough,” “dented,” “mature,” and “harvested” stages. For some states, there are additional stages, like “seedbed prepared” and “milk,” which were not used in our study. Soybean progress includes the “planted,” “emerged,” “blooming,” “setting pods,” “dropping leaves,” and “harvested” stages; stages that are only available for some states after the year 2014 were excluded. Crop progress estimates are based on survey data collected each week from numerous field enumerators, for the purposes of making field observations. Published numbers represent the percent of cropland that has reached certain stages by the end of the week.

### Variables for classification

The most important set of input variables to the classification algorithm is phenological metrics. These metrics include dates of phenological transition stages, such as emergence, mature and senescence, and the rate of vegetative development, which is useful to classify crops with different crop calendars^23^. We also used phenological metrics to interpolate MCD43A4 bands and indices from original observation dates to certain phenological stages, in order to derive “phenology-specific” multispectral variables. In this way, crop progress in different years could be “aligned” to reduce the effect of phenological variability on multispectral observations, facilitating interannual comparison.

Crop phenology was quantified by fitting pre-defined curves to time series of crop growth, characterized by the enhanced vegetation index (EVI). The EVI is relatively sensitive to crop growth during high-biomass periods, which to some extent avoids the saturation problem. The approach of curve fitting using the asymmetric double sigmoid function is similar to the phenology-based classification in an earlier paper^11^. All input variables were either directly from curve parameters or calculated from those parameters and MCD43A4 bands. Table 1 lists all input variables used in the classification and their corresponding physical meanings. When we explore the possibility of early mapping (immediately after crops reach peak growth), the time series may only include the increasing segment of the EVI profile. In this case, fitting parameters dealing with the decreasing segment would be unreasonable and must be eliminated from the input variable set. These are labeled as “late” variables in Table 1, and our selective use is explained in later sections.

A variety of spectral variables including reflectance of certain bands and multiband indices were developed with specific phenological stages or periods. These variables were selected because of their capacity to distinguish corn and soybean in numerous pilot studies for all or part of the ECB (Table 1). Each spectral variable can be further denoted by subscript “avg,” which indicates the average of the variable within the high-growth period from *D*_*i*_ to *D*_*d*_, or “peak,” which means the spectral observation interpolated to the date *D*_*peak*_ when the EVI was maximum. For example, *VI64*_*avg*_ is the average normalized difference index of MODIS bands 6 and 4 between *D*_*i*_ and *D*_*d*_. The total number of variables for full-season mapping was 27 (13 phenological metrics + 7 spectral metrics × 2 stages), and the total number for early season mapping was 21 (7 + 7 × 2).

### Classification

The random forest (RF) classifier^25^ was used for its stable performance and high efficiency to handle a large input dataset^26^,^27^,^28^,^29^,^30^,^31^. In this classifier, each tree is trained by a random subset of the original dataset, and a classification is computed by aggregating results of all tree predictors. For ECB in all study years, we conducted RF classification state-by-state and year-by-year. An RF model was trained from all pixels in a state during the training year and then applied to the mapping year for the same state.

An RF classifier only requires two parameters to generate a prediction model, i.e., the number of classification trees desired (*k*) and number of prediction variables (*m*) used in each node to grow the tree. When the value of *k* increases, the overall accuracy converges without any over-training problem^25^. Our tests indicated that the classification accuracy was not very sensitive to *k* when the RF size was sufficiently large and *k* was arbitrarily set to 100. We also found that *m* had little influence on the classification accuracy of our data, so it was set to 1 to reduce computational cost^32^. We did not focus on the influence of classifier parameters on the classification. Instead, classification results were more sensitive to options regarding input data processing, training year selection, and other factors. For a comparative analysis, we performed RF classification with every combination of these options, as described in the following sections.

#### Hard vs. fuzzy classification

For the ECB, many studies selected “pure” MODIS pixels and performed hard classification^7^,^9^. In resultant maps, discrete class labels were assigned to MODIS pixels. When finer resolution reference data are available, it is also possible to derive a continuous component cover to run a fuzzy classification algorithm that uses valuable information of crop coverage within mixed pixels. The current study compared results between the hard and fuzzy classification algorithms. First, 30-m CDL pixel footprints were overlaid on the 500-m footprints of MCD43. A coverage percentage was calculated for each MCD43 pixel by dividing the number of CDL pixels of a certain type (corn or soybean) by the total number of CDL pixels within the footprint of a MCD43 pixel, multiplied by 100. Coverage percentages of corn and soybeans were directly used in fuzzy classification. To train the hard classification algorithm, “pure” corn and soybean pixels were selected by a percentage threshold of 75 (i.e., >75% of a 500-m pixel was covered by 30-m corn or soybean pixels). This threshold was determined by examining numerous typical croplands and analyzing the influence of threshold change on crop distribution throughout the ECB^9^,^33^. An RF package in MATLAB was used to do hard classification and regression for a single class at a time as fuzzy classification^34^.

#### Full season vs. early season

We used MCD43 images from the entire year that covered the entire growing season of corn and soybeans to derive variables input to the classification algorithm. We also tested the possibility of completing crop mapping early (immediately after crops reach peak growth) without dramatically reducing the accuracy. To conduct early season classification, we selected the last day of the 28^th^ MCD43 product (8-day interval) in a year as the ending date (DOY 224, corresponding to 12 August, or 11 August in leap years), and all images after that were assumed to be “unknown”. Only images before the ending date were treated by the curve-fitting and phenology derivation procedures. By the selected date, corn and soybeans have reached their peak growth stage, with large EVI values in most ECB areas. This enables accurate phenology retrieval for the increasing segment of the EVI temporal profile.

For classification using images of the full season, all variables in Table 1 were used. By mid-August, phenological metrics regarding the decreasing segment after peak EVI are not available yet, or are derived with poor accuracy because of a limited number of observations. These metrics, labeled as late in the 3^rd^ column of Table 1, were excluded from the early-season classification.

#### Phenological similarity

We also tested if the mapping accuracy could be improved by considering phenological similarity between the training and mapping year. Although still rare in large-area crop mapping, the concept of phenological similarity or phenological synchronization have been used in multiyear applications such as yield monitoring by the MARS operational^35^,^36^ and the retrieval of canopy parameters based on thermal time^37^. For cross-year classification, the accuracy is likely to be improved when the training year and mapping year have a similar distribution of phenological metrics. The analysis was done at state level to precisely capture the phenological characteristics of each sub-region within the vast ECB. For each individual state, selected corn and soybean progress stages (all those available from the corn stages planted, emerged, silking, dough, dented, mature and harvested, and soybean stages planted, emerged, blooming, setting pods, dropping leaves and harvested) from USDA weekly reports were processed to measure interannual similarity. First, the DOY with 50% progress of each stage was derived by interpolation. Then the root-mean-square deviation (RMSD) of all 50% progress DOYs was calculated for each pair of years:


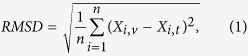


where *X*_*i,v*_ and *X*_*i,t*_ denote the 50% dates for progress item *i* in mapping year *v* and training year *t*, respectively. To facilitate cross-year comparison, original crop progress percentages from CPCR were unified to 50% and the corresponding 50% dates were calculated by interpolation. For a mapping year, the year with the smallest RMSD was considered the most similar year in terms of crop progress. All selected training years are listed in Table 2 by state. The crop progress reporting program is new in some states at the ECB boundary, such as Oklahoma and Texas, so statistics in those states were not published in earlier years because of limited area/production for corn/soybeans. The training years of these states were selected using crop progress in a neighboring state. Because of the relatively small cropping area in these states, this approximation is acceptable.

As a control group, we also used the year closest to the mapping year as the source of training data, ignoring their phenological dissimilarity. In practice, choosing the closest year for training is common and intuitively straightforward for minimizing long-term changes in climate, cropping practices, crop varieties and other factors. For a mapping year after 2009, the corresponding training year was set to the prior year. The first year in the study period was 2008, and 2009 was used as the training year for mapping year 2008 in cross-year validation. This “close year” strategy for training data selection does not distinguish individual states and therefore does not require USDA crop progress information, and has been commonly used in most earlier studies.

#### Adjustment based on crop progress

When crop phenology of the training year differs from the mapping year, classification errors are likely, because of the different distribution of phenological metrics between the two years. Classification results are less reliable when the magnitude of phenological deviation increases. Intuitively, it may be a viable means to “adjust” one year’s phenology to another by matching the distribution of phenological metrics. In the current study, we tried two simple means of phenological adjustment, which reduce interannual differences in crop progress by using USDA weekly progress data. Although there are many algorithms for histogram matching between two multi-dimensional datasets, we only used the two simple means to avoid overfitting the model and overcomplicating the process.

The first means of adjustment is to linearly correlate 50% progress dates between the two years. Phenological metrics representing phenological transition dates (labeled as date in the 4^th^ column of Table 1) were transformed from the mapping to training year using a linear relationship developed from USDA crop progress data:


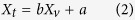


where *X*_*v*_ and *X*_*t*_ denote 50% progress dates in the mapping year *v* and the training year *t*, respectively. For each state, the slope *b* and intercept *a* from the regression equation were calculated for the cross-year transformation of phenology. For all states, the linear relationship was strong, with most coefficients of correlation > 0.99 and only a few ~0.98. Because phenological transition dates were adjusted, it was also necessary to adjust metrics that are calculated as differences between dates like growing season length (“length” in the 4^th^ column of Table 1), by multiplying these metrics by *b.* In addition, phenological metrics that represent the growth rate of crops (“rate” in the 4^th^ column of Table 1) must be divided by *b*. This type of cross-year phenological adjustment was termed “A1”.

The second adjustment, which is even simpler, is to use Equation (2) while assuming *b* = 1. This is equivalent to “shifting” one year’s phenological transition dates to the other year using the arithmetic mean of all 50% progress dates. This type of adjustment is termed “A2”. As a control group, experiments were also run without any cross-year phenological adjustment, termed “A0”.

### Validation

In our experiment, we tested all combinations of classification options above to evaluate the effectiveness of individual options, including pixel definition (hard or fuzzy), timing of input data (full or early season), choice of training year (the year closest to the mapping year, or the year with crop progress most similar to that year), and the type of adjustment of phenological metrics (no adjustment “A0”, linear fit “A1,” or simple shift “A2”). For each run of classification, the resultant corn and soybean maps, which included results of all ECB states, were compared with county statistics summarized from the USDA CDL. USDA NASS agricultural statistics are the authoritative data source for cropped area; however, we used cropped area from the CDL for validation, to be consistent with the training set. The validation was based on two quantitative measurements, the county-level coefficient of determination (*R*^*2*^) and the percentage difference of total cropped area; these were evaluated for corn and soybeans, respectively. *R*^*2*^ indicates the agreement of spatial distribution across over 2,000 ECB counties between the classification map and reference data (CDL), and the percentage difference provides an overall estimate of classification deviation, which is interesting for practical uses. The comparison of experiments with different options was based on those two measurements.

## Results

### Comparative analysis for optimum classification options

**Figure 2:**
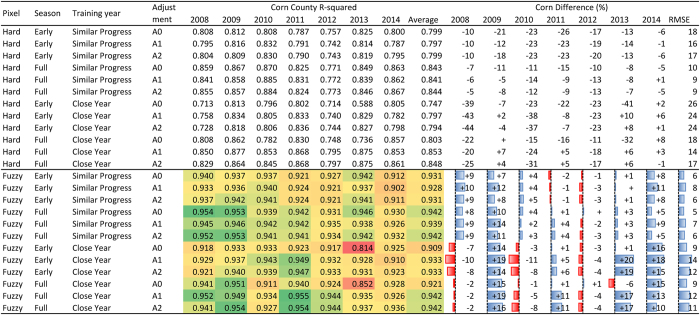
Classification results of corn with various combinations of options. The difference between fuzzy classification experiments is highlighted in color. Large R^2^ values are in green and small ones in red.

**Figure 3:**
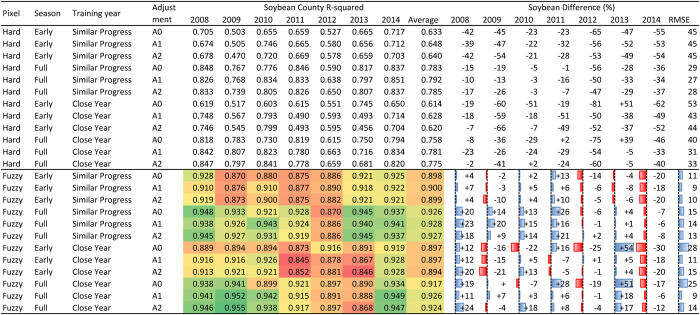
Classification results of soybeans with various combinations of options. The difference between fuzzy classification experiments is highlighted in color. Large R^2^ values are in green and small ones in red.

**Figure 4:**
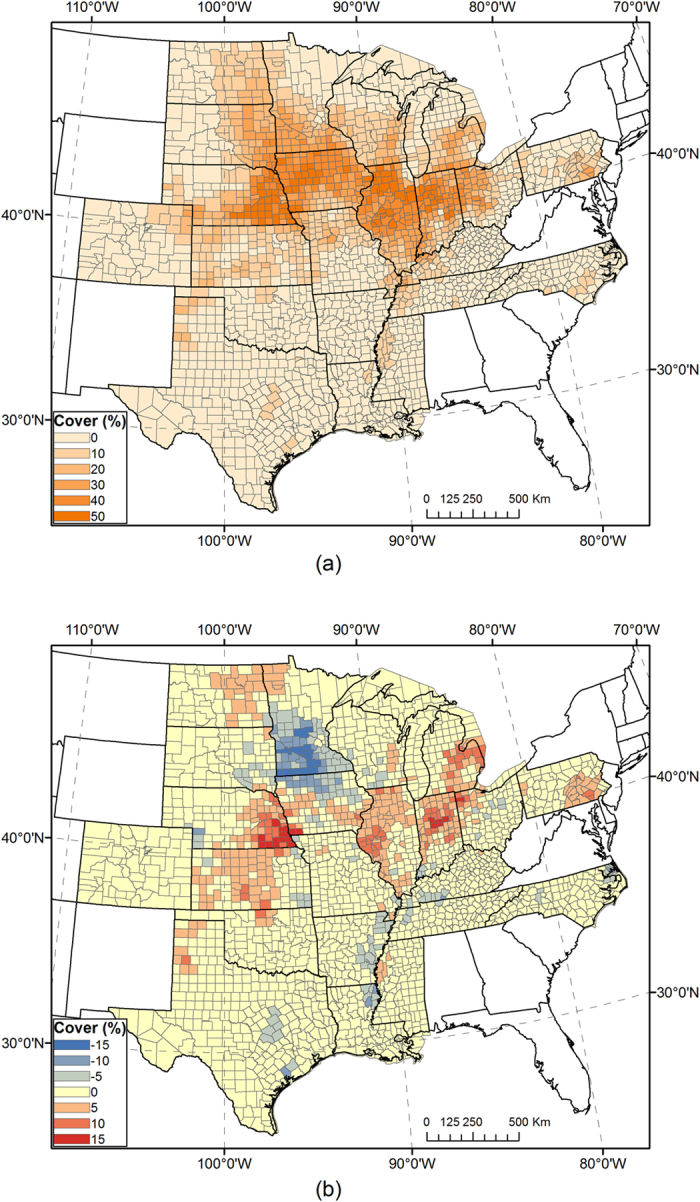
(**a**) Full-season map of corn coverage by county in 2014, using classifiers trained in 2008–2013. County-level corn coverage in percent is aggregated from per-pixel values. (**b**) Mapped county-level corn coverage minus corn coverage from the CDL. Maps were generated by ArcGIS 10.3 software (http://www.esri.com/software/arcgis/arcgis-for-desktop).

**Figure 5:**
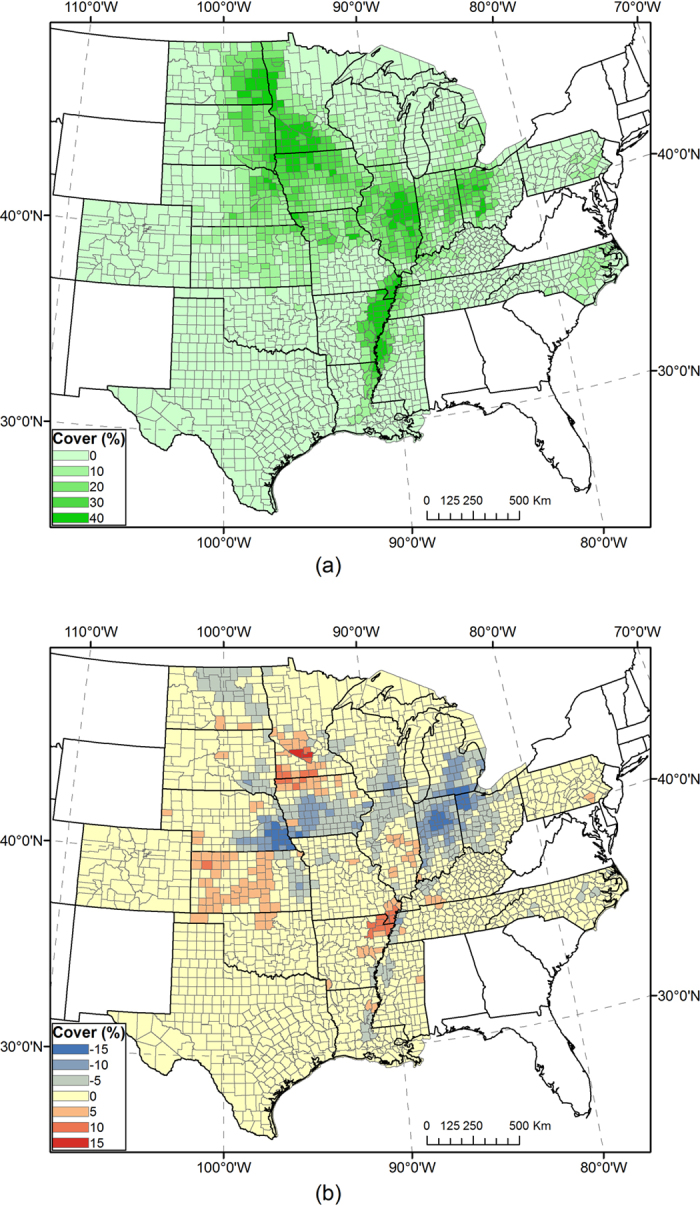
(**a**) Full-season map of soybean coverage by county in 2014 using classifiers trained in 2008–2013. County-level soybean coverage in percent is aggregated from per-pixel values. (**b**) Mapped county-level soybean coverage minus soybean coverage from the CDL. Maps were generated by ArcGIS 10.3 software (http://www.esri.com/software/arcgis/arcgis-for-desktop).

Resultant maps of all classification runs were compared with the USDA CDL in corresponding years. Visual assessment suggests that all combinations of classification options yielded reasonable spatial distributions of corn and soybeans that followed the actual pattern in the ECB. For quantitative comparison, we evaluated values of county-level *R*^*2*^ and the percentage difference of total cropped area in individual mapping years for corn and soybeans, respectively (Figs 2 and 3). For example, the corn map in 2014 using classification options“fuzzy,” “full season,” “similar progress,” and “no adjustment (A0)” achieved an *R*^*2*^ value of 0.930, and total corn area in the study area was overestimated by 5% compared with the CDL. The corresponding soybean map has *R*^*2*^ = 0.937 and bias = −7%. County-level maps of crop cover and difference from the CDL for corn and soybeans are presented in Figs 4 and 5, respectively, which indicate the county-level distribution of mapped crop extent and mapping uncertainty. In general, there are many large *R*^*2*^ values (>0.9) in the figures, indicating strong agreement considering the large number of counties in the ECB (>2,000). For detailed comparison, maps of all classification options and years as well as the two measurements of individual states were also inspected, which could not be presented here for brevity. We calculated the arithmetic mean of *R*^*2*^ and RMSE of percentage difference in all mapping years, to estimate the general performance of a classification run.

The results suggest that a fuzzy classifier is superior to hard classification. All fuzzy runs consistently yielded much larger *R*^*2*^ for both corn and soybeans than their counterparts from the hard classification. This is expected, because use of the high-quality dense time series from MCD43 as the main input is at the cost of relatively low spatial resolution. The 500-m resolution may be sufficient for classifying most large-scale crops in the ECB, but there remain many mixed pixels. When assigning discrete types to pixels by using threshold values of coverage percentage, loss of information is inevitable. The limitation of hard classification when using MODIS products in an agricultural system similar to the ECB has been reported in other studies^7^,^9^. Therefore, it is a must to consider mixed pixels and use a fuzzy classification method for our study area and input data. Thus, the following analysis focuses on fuzzy classification runs only.

For other classification options, resultant *R*^*2*^ and percentage difference values are shown by color shading in Fig. 2 to highlight the difference in performance. Full-season classification consistently achieved better agreement with reference data than early-season, which is expected because input data of early-season classification is only a subset of full-season variables. For corn, the difference is very small, suggesting that the timing of corn mapping could be advanced to the middle of the growing season without sacrificing much accuracy. The difference in agreement of soybean mapping is slightly larger. Since soybean has later progress and peak growth dates than corn, the possible reason for such disagreement is that by the date of early-season classification, a portion of soybeans has not reached the high biomass stage. In southern states like Illinois, early-season and full-season soybean mapping gave very similar results and overall area estimates. In northern states like North Dakota where crop progress is later, the advantage of full-season classification is more noticeable.

For the choice of training year, the year selected based on crop progress similarity (similar progress) gave less bias than the year closest to the mapping year (close year). In terms of RMSE of percentage difference in all years, all runs of corn classification with the similar-progress option resulted in RMSE between 5% and 8%, and the close-year option produced 9% to 14%. For soybeans, corresponding ranges of the two options were 9–15% and 9–28%, respectively. Unlike the close-year option which in some years yielded very small *R*^*2*^ values (below or around 0.85), the selection of training year based on crop progress showed very stable performance, as indicated by more consistent *R*^*2*^ values. This suggests that the use of USDA crop progress data may reduce uncertainty caused by the phenological deviation between the mapping and training year. It is definitely necessary to consider interannual variability in crop phenology in cross-year classification and validation.

When all other classification options were the same, the choice of adjustment method did not have much impact on the results. Given the very complex distribution of phenological metrics in various years and areas, our simple adjustment methods (linear regression or shift) are likely to be insufficient to match the phenology. Although for most classification runs the difference is trivial, there are a few instances when adjustments play an effective role. Upon choosing 2012 as the training year for mapping year 2013 using the “close year” option, *R*^*2*^ values of corn classification were very small, 0.814 for the early season and 0.852 for the full season. In most ECB areas, 2012 showed the earliest crop progress, and 2013 was one of the latest years. In this case, both adjustment methods noticeably improved county-level agreement for corn, suggesting the utility of phenological adjustment between the two years with distinct crop progress. When the training year was selected based on similar progress, phenology between the training and the mapping year was usually very similar, making it unnecessary in most cases to further adjust and match phenology. In addition, the effect of phenological adjustment was usually different between corn and soybeans, implying that it is challenging to adjust the phenology of both crops at the same time.

Considering the reliability and stability of the classification results and the simplicity of algorithm implementation, the most effective combination of classification options was selected as fuzzy, full season, similar progress, and no adjustment (A0). We are also interested in the optimum classification procedure in the middle of the growing season for the practical need of early mapping (selecting “early season”), which was determined as fuzzy, similar progress, or no adjustment (A0). These options will continue to be used in full-season and early-season mapping efforts for future years, after the training period 2008–2014.

### Early-season mapping in 2015

**Figure 6:**
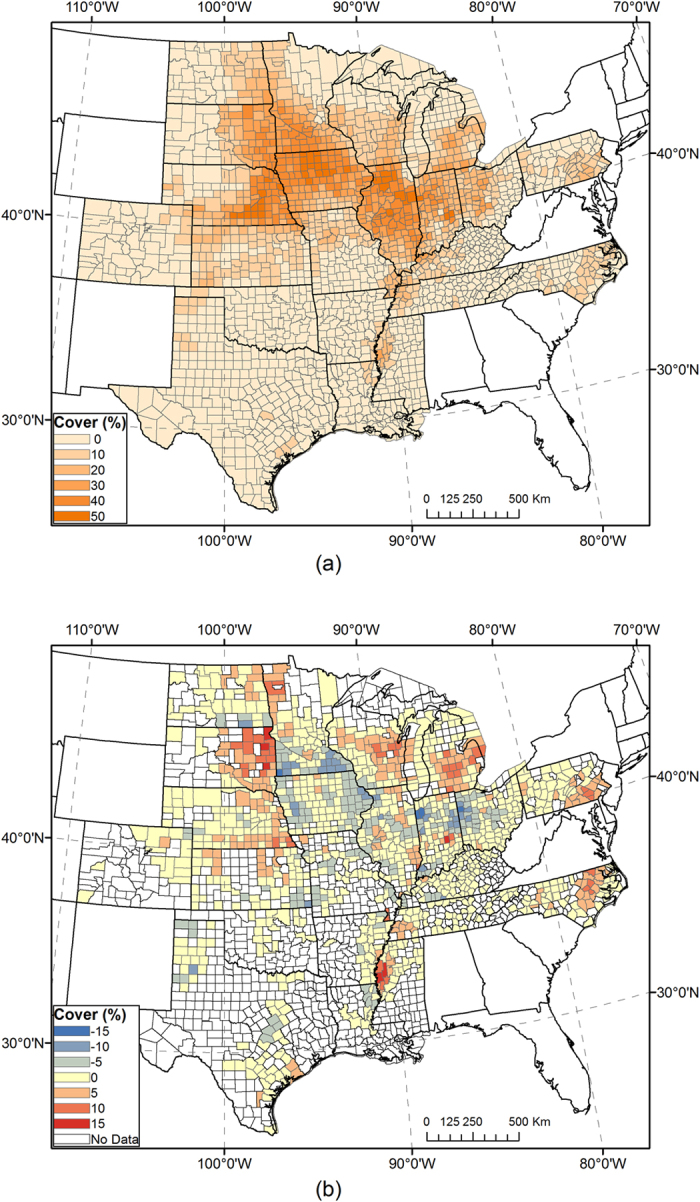
(**a**) Early-season map of corn coverage by county in 2015, using classifiers trained in 2008–2014. County-level corn coverage in percent is aggregated from per-pixel values. (**b**) Mapped county-level corn coverage minus corn coverage from USDA NASS statistics. Maps were generated by ArcGIS 10.3 software (http://www.esri.com/software/arcgis/arcgis-for-desktop).

**Figure 7:**
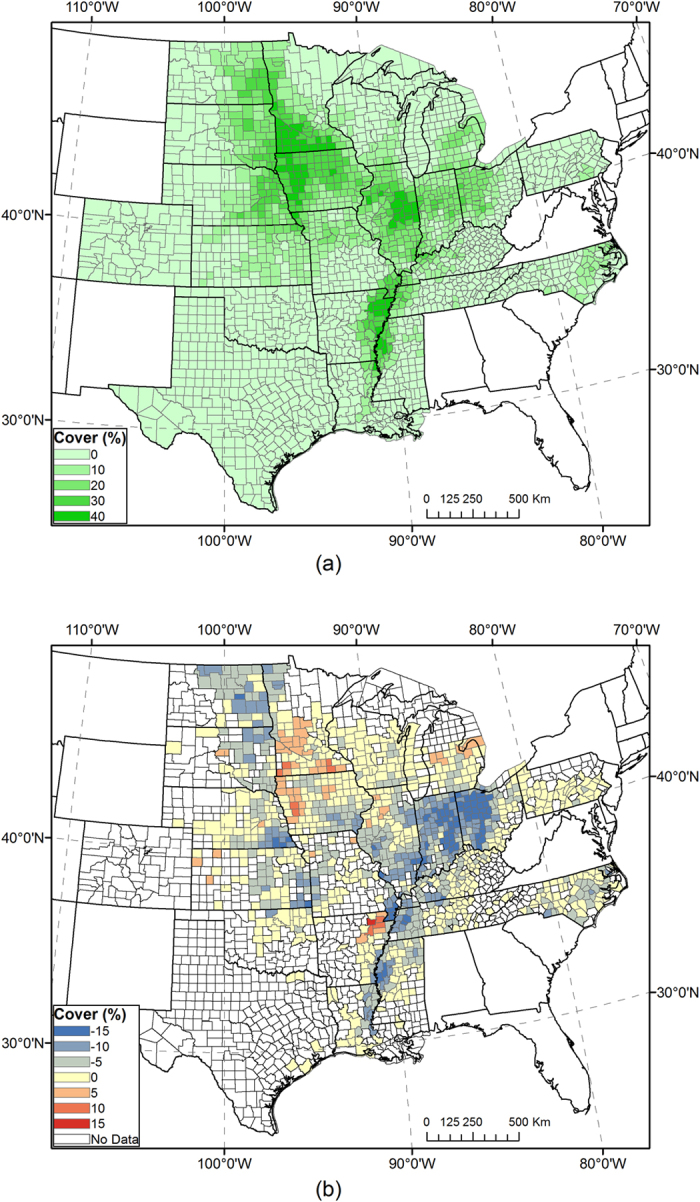
(**a**) Early-season map of soybean coverage by county in 2015, using classifiers trained in 2008–2014. County-level soybean coverage in percent is aggregated from per-pixel values. (**b**) Mapped county-level soybean coverage minus soybean coverage from USDA NASS statistics. Maps were generated by ArcGIS 10.3 software (http://www.esri.com/software/arcgis/arcgis-for-desktop).

We conducted early season mapping for the year 2015 using RF classification models trained in 2008–2014. Classification options were selected based on cross-year validation results from that period, as described in the previous section. Resultant corn and soybean coverage maps show spatial patterns of crop cultivation in ECB states (Figs 6 and 7). By the time of our study, the 2015 CDL was still unavailable. Both maps were compared with USDA official statistics of individual counties. County level *R*^*2*^ was 0.956 for corn and 0.894 for soybeans. The total acreage of corn was underestimated by 1.9%, and soybeans by 17.6%. These measurements suggest that the accuracy of 2015 early season maps is similar to the cross-year validation in 2008–2014, but slightly lower than most years. It should be noted that the CDL, a remote sensing-based classification map, always differs from field survey-based statistics, although the self-reported accuracy of the CDL is consistently high^8^. Because classifiers in our study were trained by the USDA CDL, we expect our classification maps to resemble the CDL more than the USDA statistical data pattern.

## Discussion

### Early-season mapping and cross-year validation

The uniqueness of this study is (i) the development of current-year crop maps based on classification algorithms previously trained with historical reference data, and (ii) quantitative measurement of phenological similarity between years to select optimum training year. The cross-year validation may greatly expedite mapping by overcoming the difficulty of collecting training data for a large area within the current growing season. Because of differences in weather, agricultural management and other factors, crop progress varies by year, leading to inconsistent temporal profiles of remote sensing observations. Our experiments show that by quantitatively accounting for the interannual variability of phenology, it is possible to align crop progress stages across years to minimize the effect of phenological changes.

In remote sensing classification studies, actual data distributions of the training set and test/validation set are never identical. It is possible, however, to enhance consistency between the two sets by choosing the most similar training set or adjusting one set to match the other. The use of USDA weekly crop progress data facilitates quantitative evaluation of the phenology of all previously trained years and selection of the year with the most similar phenology. This is presumed to minimize the difference between the training and mapping years, toward high-accuracy mapping. However, it is inevitable that the selected training year differs in phenology from the mapping year, especially when there are only a few trained years as choices. As time goes by, more and more years with available reference data (in this case, the CDL) will become candidates for a training year. This forms a large pool of trained classifiers representing various conditions of weather and agricultural management, from which a suitable match is likely. The advantage of our cross-year mapping is annual classification based on a growing historical record of trained classifiers, which has the potential of continuously improving mapping accuracy year by year.

### Sources of uncertainty

**Figure 8:**
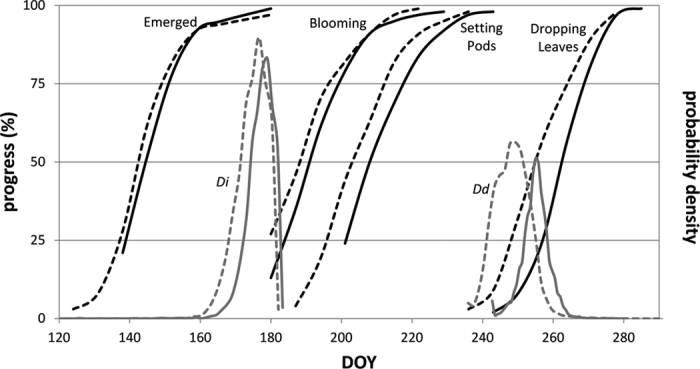
USDA weekly soybean progress (black) and histogram of metrics *D*_*i*_ and *D*_*d*_ derived from EVI profiles of pure soybean pixels (grey), for state of Iowa in 2010 (dashed lines) and 2011 (solid lines).

Based on our observations, the curve-fitting approach successfully retrieved phenology-related metrics and quantified inter-class and spatial variability of phenology. An example is given in Fig. 8, in which USDA percentage weekly soybean progress in Iowa is denoted by black lines and histograms of metrics *D*_*i*_ and *D*_*d*_ in gray. In 2010 and 2011, the remote sensing-derived phenological measurements were very consistent with the official survey-based crop progress data. Around early stages like emerged and blooming, the difference between 2010 and 2011 was small. Slightly earlier progress in 2010 is reflected by the histogram of *D*_*i*_. The difference is increasingly large as soybean grew in later stages. Both the weekly progress of dropping leaves and the histogram of *D*_*d*_ suggest that 2010 was about 10 days earlier, when soybean biomass declined. Therefore, although 2010 was generally the most similar year to 2011 in terms of phenology or crop progress, there were some large differences in certain stages of the growing season. As a result, when the 2011 early-season soybean map was created using classifiers trained in 2010, soybean area was overestimated by 35%, the largest error in the study, because many corn fields with slightly earlier phenology than soybeans were misclassified as soybeans using the earlier crop progress in 2010 as the reference. The small overall phenological difference between 2010 and 2011, simply calculated as the RMSE of all 50% progress dates, may have underestimated the partial-season difference in progress. Through adjustments A1 (linear fit) and A2 (simple shift), the error was reduced to 15% and 16%, respectively. However, the simple adjustment methods used may still be unable to effectively handle the complex seasonal pattern of phenology.

**Figure 9:**
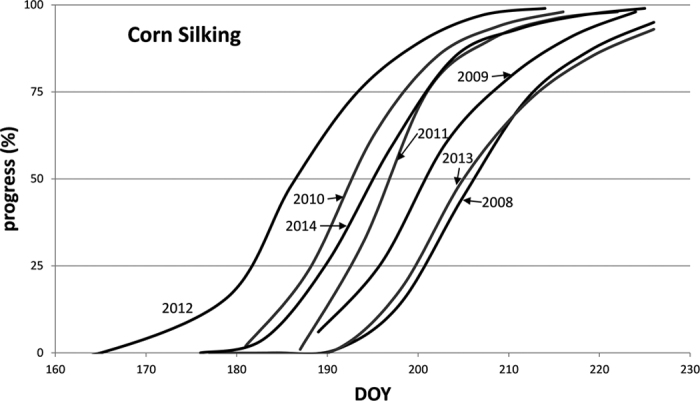
USDA weekly progress of corn silking for state of Iowa in 2008–2014.

Corn mapping is also affected by interannual variability in crop progress. For example, corn silking is a critical progress stage during the peak growth period. The 50% date of corn silking was subject to about 20 days’ difference in Iowa during 2008–2014 (Fig. 9). Such a difference is comparable to, if not larger than, the inter-class difference between corn and soybeans, especially for years with very early or very late phenological development. When a year was selected for training based on overall progress throughout the season, the training and mapping years could still have different phenology during certain stages. For Iowa, 2014 was considered the year most similar to 2009 in terms of overall phenology (Table 2), but their difference in the corn silking stage is apparent, which might have caused the low accuracy in 2009 mapping (county level *R*^*2*^ = 0.880, the smallest for early-season corn mapping of Iowa). When years with very early or very late crop development are involved, inaccurate classification is more likely. The year 2012 had a record pace of planting and crop development because of very high temperatures near the beginning of the growing season. However, later crop development was severely impacted by an unprecedented drought, caused by high temperatures combined with scarce rainfall. Crop growth in 2012 was unique and not comparable to any recent year. By contrast, 2013 had very late planting dates because of excessive rains during the normal planting season. As a result, when mapping 2013 crop extent, it was very problematic to use trained classifiers from the previous year 2012. Instead, classifiers trained with 2008 data were used to represent the late growing conditions. Because the extremely late crop development in 2013 was caused by excessive precipitation, phenological abnormality may not have been consistent across regions, but USDA weekly crop progress data are only available for large spatial units such as states. Given all sources of complexity in cross-year phenology comparison, we see that adjustments by linear matching (A1) or simple shift (A2) may still be inadequate to eliminate differences in the distribution of phenological metrics.

Regarding the selection of training years, we conclude that a year with similar crop progress is superior to a year close to the mapping period. However, errors caused by interannual phenological variability have not been fully addressed. The ultimate solution, according to the discussion in the previous section, is to maintain a long historical record of training years from which it is always possible to find a year with phenology sufficiently similar to the mapping year. Before such historical data are available, potential solutions include (i) using more complicated, nonlinear or weighted adjustment approaches to match one year’s phenology to another, and (ii) developing more delicate quantitative measurements of crop progress in a specific year rather than simply using all 50% progress dates. In general, the magnitude of interannual variability in crop progress or phenology is always an important factor to consider in cross-year mapping/validation.

## Conclusions

In the present study, we developed a rapid mapping approach for corn and soybeans in the US Corn Belt and adjacent states. Traditionally, the development of timely crop maps of a large area is limited by the tremendous amount of time required by reference data collection. This makes it extremely difficult to finish mapping within the current season. Our approach greatly accelerates the mapping process by using trained classifiers that have been prepared in advance, using reference data from other years instead of relying on current-year ground reference data. Cross-year validation in 2008–2014 showed that the rapid mapping is capable of producing timely and accurate results when using fuzzy classification and phenology-based training year selection. For the middle of the 2015 growing season, we successfully produced corn and soybean coverage maps using trained models selected from 2008–2014, according to phenological similarity. The study also establishes a framework to maintain a historical record of trained classification models for prompt year-by-year mapping in the future. As the record grows longer and covers a diversity of crop progress conditions with an increasing number of trained models, the impact of interannual phenological variability will be reduced and the robustness of resulting map products will be continuously improved. The framework is also applicable to medium-resolution imagery from missions such as Landsat and Sentinel, if the image product is properly standardized.

## Additional Information

**How to cite this article**: Zhong, L. *et al*. Rapid corn and soybean mapping in US Corn Belt and neighboring areas. *Sci. Rep.* **6**, 36240; doi: 10.1038/srep36240 (2016).

**Publisher’s note:** Springer Nature remains neutral with regard to jurisdictional claims in published maps and institutional affiliations.

## Figures and Tables

**Table 1:** Phenological and other variables generated as input to the classification algorithm.

Variable	Description	Season	Type of adjustment
Phenological metrics from EVI time series and curve-fitting parameters:			
*V*_*b*_	“Background” EVI value corresponding to non-growing season.		
*V*_*a*_	Amplitude of EVI variation within the growing cycle.		
*p*	Slope parameter of the increasing segment in the cycle.		Rate
*D*_*i*_	Middle date of the increasing segment with maximum first derivative.		Date
*q*	Slope parameter of the decreasing segment.	Late	Rate
*D*_*d*_	Middle date of the decreasing segment with minimum first derivative.	Late	Date
*D*_*1*_	Date with local maximum second derivative when EVI starts increasing.		Date
*D*_*2*_	Date with local minimum second derivative when EVI stops increasing.		Date
*D*_*3*_	Date with local minimum second derivative when EVI starts decreasing.	Late	Date
*D*_*4*_	Date with local maximum second derivative when EVI stops decreasing.	Late	Date
*L*_*id*_	Difference between *D*_*i*_ and *D*_*d*_, representing growing season length.	Late	Length
*L*_*14*_	Difference between *D*_*4*_ and *D*_*1*_, representing growing season length.	Late	Length
*D*_*peak*_	Date with maximum EVI.		Date
Spectral metrics to be combined with phenological stages:			
*SWIR1*	MODIS band 6 (1628–1652 nm, shortwave infrared) reflectance.		
*SWIR2*	MODIS band 7 (2105–2155 nm, shortwave infrared) reflectance.		
*EVI*	EVI.		
*NDTI*	Normalized Difference Tillage Index^24^.		
*VI64*	Normalized difference index of band 6 and band 4 (545–565 nm).		
*DIF64*	Band 6 reflectance minus band 4 reflectance.		
*DIF67*	Band 6 reflectance minus band 7 reflectance.		
Subscripts of spectral metrics to indicate phenological stages:			
*avg*	Average between *D*_*i*_ and *D*_*d*_, or after *D*_*i*_ if *D*_*d*_ is not yet available.		
*peak*	Value at date *D*_*peak*_.		

**Table 2:** Selected training years for all mapping years based on phenological similarity.

State	Mapping year							
	2008	2009	2010	2011	2012	2013	2014	2015
Arkansas	2013	2008	2014	2013	2010	2011	2010	2010
Colorado	2010	2011	2008	2009	2010	2008	2011	2008
Illinois	2013	2008	2012	2014	2010	2008	2011	2011
Indiana	2013	2011	2012	2008	2010	2008	2013	2013
Iowa	2013	2014	2011	2010	2010	2008	2011	2011
Kansas	2013	2008	2011	2010	2010	2008	2011	2010
Kentucky	2011	2013	2012	2008	2010	2009	2008	2014
Louisiana	2009	2010	2009	2013^a^	2014	2009	2012	2010
Michigan	2013	2014	2012	2014	2008	2008	2011	2008
Minnesota	2013	2008	2012	2014	2010	2008	2008	2010
Mississippi	2011	2008	2014^a^	2014	2014	2014	2011	2014
Missouri	2013	2008	2011	2010	2010	2008	2010	2011
Nebraska	2013	2014	2011	2013	2010	2011	2010	2010
North Carolina	2012	2011	2011	2009	2008	2008	2011	2014
North Dakota	2014	2008	2011	2014	2010	2014	2013	2010
Ohio	2013	2014	2012	2009	2010	2008	2009	2013
Oklahoma	2013^b^	2008^b^	2011^b^	2010^b^	2010^b^	2008^b^	2011^b^	2014
Pennsylvania	2013	2014	2012	2014	2010	2008	2009	2012
South Dakota	2013	2008	2013	2010	2010	2010	2013	2010
Tennessee	2014	2014	2012	2008	2010	2009	2008	2008
Texas	2013^b^	2008^b^	2011^b^	2010^b^	2010^b^	2008^b^	2011^b^	2014
Wisconsin	2014	2014	2012	2014	2010	2008	2008	2012

Mapping years are 2008–2015 and training years 2008–2014. There was no reference data available for 2015 and this was used only for validation.

^a^Crop progress data are not complete. Used the same selection as neighboring state Arkansas.

^b^Crop progress reporting has not begun. Used the same selection as neighboring state Kansas.
